# Prognostic Utility of Multivariate Morphometry in Schizophrenia

**DOI:** 10.3389/fpsyt.2019.00245

**Published:** 2019-04-15

**Authors:** Mingli Li, Xiaojing Li, Tushar Kanti Das, Wei Deng, Yinfei Li, Liansheng Zhao, Xiaohong Ma, Yingcheng Wang, Hua Yu, Yajing Meng, Qiang Wang, Lena Palaniyappan, Tao Li

**Affiliations:** ^1^Mental Health Center and Psychiatric Laboratory, State Key Laboratory of Biotherapy, West China Hospital of Sichuan University, Chengdu, China; ^2^West China Brain Research Center, West China Hospital of Sichuan University, Chengdu, China; ^3^Robarts Research Institute and The Brain and Mind Institute, University of Western Ontario, London, ON, Canada; ^4^Department of Psychiatry, University of Western Ontario, London, ON, Canada; ^5^Lawson Health Research Institute, London, ON, Canada

**Keywords:** schizophrenia, longitudinal, gray matter, multivariate morphometry, predictor

## Abstract

**Background:** Voxel-based morphometry studies have repeatedly highlighted the presence of distributed gray matter changes in schizophrenia, but to date, it is not clear if clinically useful prognostic information can be gleaned from structural imaging. The suspected association between gray matter volume (GMV) and duration of psychotic illness, antipsychotic exposure, and symptom severity also limits the prognostic utility of morphometry. We address the question of whether morphometric information from patients with drug-naive first-episode psychosis can predict the linear trajectory of symptoms following early antipsychotic intervention using a longitudinal design.

**Method:** Sixty-two first-episode, drug-naive patients with schizophrenia underwent brain magnetic resonance imaging scans at baseline, treated with antipsychotics, and rescanned after 1-year follow-up. Positive and Negative Syndrome Scale (PANSS) was used to assess their clinical manifestations. A multivariate approach to detect covariance-based network-like spatial components [Source Based Morphometry (SBM)] was performed to analyze the GMV. Paired *t* tests were used to study changes in the loading coefficients of GMV in the spatial components between two time points. The reduction in PANSS scores between the baseline (T0) and 1-year follow-up (T1) expressed as a ratio of the baseline scores (reduction ratio) was computed for positive, negative, and disorganization symptoms. Separate multiple regression analyses were conducted to predict the longitudinal change in symptoms (treatment response) using the loading coefficients of spatial components that differed between T0 and T1 with age, gender, duration of illness, and antipsychotic dose as covariates. We also tested the putative “toxicity” effects of baseline symptom severity on the GMV at 1 year using multiple regression analysis.

**Results:** Of the 30 spatial components of gray matter extracted using SBM, loading coefficients of anterior cingulate cortex (ACC), insula and inferior frontal gyrus (IFG), superior temporal gyrus (STG), middle temporal gyrus (MTG), precuenus, and dorsolateral prefrontal cortex (DLPFC) reduced with time in patients. Specifically, the lower volume of insula and IFG at baseline predicted a lack of improvement in positive and disorganization symptoms. None of the symptom severity scores (positive, negative, or disorganization) at baseline independently predicted the reduced GMV at 1 year.

**Conclusions:** The baseline deficit in a covariance-based network-like spatial component comprising of insula and IFG is predictive of the clinical course of schizophrenia. We do not find any evidence to support the notion of symptoms per se being neurotoxic to gray matter tissue. If judiciously combined with other available predictors of clinical outcome, multivariate morphometric information can improve our ability to predict prognosis in schizophrenia.

## Introduction

Over the last five decades, several neuroimaging studies have reported numerous morphological abnormalities in the brain, especially in the gray matter volume (GMV), in patients with schizophrenia. Meta-analytical syntheses of whole-brain voxel-based morphometric studies have found consistent reduction in gray matter volume (GMV) of the anterior insula, anterior cingulate cortex (ACC), superior temporal gyrus (STG), middle and inferior frontal gyrus, and thalamus, even at the time of first-episode psychosis (1, 2). Such repeated observations have raised the promise of morphometric signatures being utilized as biomarkers for clinical use (3, 4), though, to date, this promise is yet to be realized (5).

The nonspecific nature of GMV deficits across various psychiatric disorders has reduced the diagnostic utility of morphometry (6, 7). Nevertheless, the pathophysiological and outcome-related heterogeneity of schizophrenia raises the prospect of using morphometric variations to predict prognosis. Given that more stable outcome patterns emerge by around 1 year of treatment in first-episode samples, a number of studies have focused on identifying the structural determinants of 1-year outcome (8). These studies have provided promising leads that are nevertheless isolated observations that are nonreplicated to date, variously implicating striatum (9), lateral ventricles (10, 11), parahippocampal cortex (12), and dorsolateral prefrontal cortex (13, 14). Some studies have observed no relationship between symptom change over 1 year and changes in brain structure (10, 15).

The limitations contributing to the inconsistency in linking morphological features to clinical outcome at 1 year includes 1) the use of univariate approaches applied to selected brain regional volume measurements, 2) the lack of drug-naive samples at the baseline, 3) the lack of longitudinal MRI data to address the issue of reverse association, i.e., the effect of symptom severity and antipsychotic dosage (15–17) on subsequent gray matter reduction, and 4) the use of relatively modest samples (median sample size of the previous seven studies, *n* = 39).

Voxel-based analysis (VBM) has been the most common method used to date to locate GMV changes in schizophrenia, but this mass univariate approach fails to take into account the relationship (covariance) among brain regions introduced by maturational and neuroplastic processes as well as intrinsic connectivity (“common fate”). Source-based morphometry (SBM) is a multivariate extension of VBM with independent component analysis for identifying naturally grouping, maximally independent sources of GMV (18, 19). SBM thus identifies covariance-based morphological networks whose variation among individuals can be exploited to symptom-related variations (20). This approach also could increase the sensitivity of morphometric studies by compartmentalizing noise, allowing distributed brain regions sharing “common fate” to be studied as single units, and reducing the loss of information due to conservative statistical approaches required for the meaningful use of VBM (19).

The present study recruited a relatively large sample of 62 patients with first episode schizophrenia and scanned them at antipsychotic-naive baseline and followed them for further 1 year. Our primary aim was to investigate the spatial pattern of progressive gray matter changes that predict the symptomatic treatment response after 1-year follow-up. We also examined the relationship between baseline severity of symptoms and lower gray matter volume observed after 1 year of treatment.

## Materials and Methods

### Participants

Sixty-two ﬁrst-episode and drug-naive patients with schizophrenia from the Mental Health Center in West China Hospital were enrolled in the longitudinal study and followed up at 1-year interval after their first psychotic episode. They were interviewed and assessed using the Structured Clinical Interview for the *DSM-IV* (*Diagnostic and Statistical Manual of Mental Disorders, Fourth Edition*) (SCID-I/P) (21) and fulfilled diagnostic criteria for schizophrenia or schizophreniform psychosis in the *DSM-IV*. The participants with evidence of organic brain disorders, alcohol use disorder, or operationally defined “drug abuse,” or any other severe physical illness such as brain tumor or epilepsy were excluded. The “drug abuse” definition was based on the drug list in SCID-I including sedatives, cannabis, stimulants, opioids, cocaine, hallucinogen, phencyclidine, and others (e.g., steroids, diet pills).

All participants were Han Chinese and right-handed. The handedness of the participants was assessed with Annett Handedness Scale (22). This study was carried out in accordance with the Declaration of Helsinki and was approved by the Institutional Review Board of West China Hospital, Sichuan University. After a complete description of the study to the participants, written informed consent was obtained.

The severities of symptoms and social functional damage were evaluated by two experienced psychiatrists (ML and WD) using positive and negative symptoms scale [Positive and Negative Syndrome Scale (PANSS)] (23) both at baseline and 1 year after (overall inter-rater reliability ICC = 0.91).

We initially recruited a sample of 90 subjects, of whom 66 completed the 1-year follow-up scan; the scans of 4 subjects were discarded due to significant movement artifacts/lack of symptom change data and 62 subjects were included in the current study.

### Antipsychotic Treatment

In the present study, patients received antipsychotic medications according to the preference of the treating clinician and the patient. This followed a hospital-wide protocol of preferring atypical antipsychotics as first line, and changing the treatment depending on nonresponse or intolerance assessed periodically (2–4 weeks in most cases). The details of the longest prescribed treatment and dose were assessed from patients or their guardians’ reports and hospital records. The frequency of each type of antipsychotic is listed in **Supplementary Table 1**. All except five patients received atypical antipsychotics, predominantly risperidone (32 of 62). While the data on compliance and medication switching are not available, all 62 patients were continuously prescribed antipsychotic medications throughout the study. The dosage of antipsychotic medication taken by each patient was recorded and converted to chlorpromazine equivalent dosages using the conversion table provided by Atkins (24) and Woods (25) (**Table 1**).

**Table 1:** Demographics and clinical data.

	Patients baseline	Patients 1 year follow-up	Reductive ratio (%)^#^	df	*t* value	*p* value
Age	24.17 (8.14)			—	—	—
Educational years	12.46 (2.80)			—	—	—
Duration of illness (months)	7.96 (11.46)			—	—	—
Scan interval (months)	12.39 (0.76)					
Total CPZ equivalent dosages (mg)	—	87,943.75 (61,826.71)		—	—	—
Gender (male/female)	24/39			—	—	—
PANSS						
Positive	27.32 (5.37)	12.40 (7.04)	0.54 (0.26)	61	14.77	0.000
Negative	20.97 (7.92)	14.73 (5.83)	0.23 (0.31)	61	6.38	0.000
Disorganization	34.69 (6.37)	19.32 (8.16)	0.43 (0.24)	61	12.53	0.000

^#^Reductive ratio is calculated as ((baseline − follow up)/baseline) × 100; CPZ, chlorpromazine; PANSS, Positive and Negative Syndrome Scale, mean (SD).

### Magnetic Resonance Imaging Data Acquisition

All the participants were scanned on a Signa 3.0-T scanner (General Electric, Medical Systems, USA) at baseline and 1 year after in the Department of Radiology at West China Hospital. A water phantom was scanned to get data quality assurance indexes every time, which was used to evaluate the stability of the MRI machine. High-resolution T1 images were acquired by 3D spoiled gradient echo sequence (SPGR) as follows: repetition time, 8.5 ms; echo time, 3.93 ms; flip angle, 12°; slice thickness, 1 mm; single shot; ﬁeld of view, 24 cm × 24 cm; matrix, 256 × 256; and voxel size, 0.47 × 0.47 × 1 mm^3^. This sequence lasted 6 min and 30 s, and 156 contiguous coronal slices were collected. The quality of the brain images was examined immediately after each scan; the scans were repeated if gross distortions were found. The median interscan interval was 12.39 months (range, 11.06–14.90 months).

### Image Preprocessing

T1 images were processed using the Diffeomorphic Anatomical Registration Through Exponentiated Lie algebra (DARTEL) toolbox in Statistical Parametric Mapping (SPM) 8. The preprocessing steps are as follows (26): Firstly, both baseline and follow-up scan T1-weighted images were realigned manually according to the AC-PC line and midsagittal plane. The baseline image was manually coregistered to the follow-up scan image without reslicing. Second, all the T1-weighted images were segmented into probability maps of gray matter (GM), white matter (WM), and cerebrospinal fluid in SPM8 using DARTEL’s segmentation algorithm that uses prior information in standard space. The resulting GM and WM probability maps were resampled into 1-mm isotropic voxels. Third, a subject-speciﬁc template was created for each participant using the information of both GM and WM maps. This template was generated by combining the GM/WM maps of the baseline and follow-up scans into average GM/WM maps using an automated unbiased template building, nonlinear registration program (DARTEL). The baseline and follow-up scan GM/WM maps were then spatially normalized onto the corresponding subject-speciﬁc template. The signal intensity of the normalized images was modulated by the determinant of the Jacobian of the transformation to account for expansion and/or contraction of brain regions. Fourth, a population template was created by simultaneously registering all subject-speciﬁc GM/WM templates using DARTEL. The template-registered images of the baseline and follow-up scans of each participant were normalized to the population template and then modulated. Fifth, in order to bring the ﬁnal analysis into standard Montreal Neurological Institute (MNI) space, the population GM template was registered automatically to the MNI space through an afﬁne transformation. Thus, all the individual GM images were affine-registered to a common anatomical space. Finally, these GM images were smoothed with a 6-mm full-width, half-maximum kernel.

### Source-Based Morphometry

SBM analysis was carried out using Group ICA Toolbox as per the standard descriptions provided by the authors (18). The number of components was set as 30, consistent with an earlier study (27). The subjects-by-voxels matrix was decomposed into a mixing matrix (subjects-by-components) representing loading parameters that quantify the contribution of each subject to the group for a given component and source matrix (components-by-voxels) representing the spatially independent “networks” defined on the basis of morphometric covariance within the group as our previous study (20). We employed ICASSO (a bootstrapping algorithm for 20 iterations) to increase the stability of the estimated components (28). Artifact components were identified visually and not included in subsequent analyses. To visualize the spatial components, the source matrix was recomposed to statistical maps in three-dimensional MNI space with coefficients expressed in standard deviation units (*z*-maps). The anatomical descriptions of these maps were obtained using the Talairach coordinate utility in the GIFT toolbox.

The GMV of each voxel in each participant (after removal of the group mean) is the sum of 30 product values obtained from multiplying the voxel loading for each component with its coefficient for that subject. The sign of the loading coefficients of a component in each participant does not directly provide the direction of change in absolute GMV in a region (27). As a result, the loading coefficients of the spatial ICA GM maps need careful interpretation. A larger loading coefficient for an individual or group indicates that the spatial pattern is more strongly weighted in the data for the individuals or group, but the interpretation of the loading coefficient difference depends on the sign of the spatial component. If the spatial component is predominantly positive, the loading coefficients are lower at follow-up than at baseline; we can infer that GMV is lower at follow-up compared to the baseline. In contrast, if the spatial component is predominantly negative, with the loading coefficients being lower at follow-up than at baseline, then the GMV is increased at follow-up compared to baseline for the spatial component under consideration.

To identify artifacts, we used the criteria employed by Xu et al. in their original description of SBM (18). Components that were exclusively composed of sharp edges around the boundary of the brain/skull or occurring in regions expected to have no gray matter were excluded. On visual inspection, four such components were identified as obvious artifacts and were excluded from further analyses (**Supplementary Figure 2**).

### Statistical Analysis

#### Demographic and Clinical Characteristics

All statistical tests were carried out by SPSS, Version 19 (IBM, Armonk, New York). We were interested in the time course of symptoms (change scores) across various domains. In order to adjust for baseline symptom severity, the changes in positive, negative, disorganization factor scores from PANSS (29) between baseline and 1 year were expressed as a ratio of the respective scores at the baseline, providing baseline-adjusted reduction ratio for positive, negative, and disorganization domains.

For the GMV data from the structural MRI, paired *t* tests for loading coefficients were first employed to identify the spatial components with progressive changes. A threshold of *p* < 0.005 following Bonferroni correction was deemed to indicate statistical significance.

### Gray Matter Volume at Baseline as a Predictor of Response to Treatment

To assess whether change in symptom scores is associated with specific baseline GMV components in schizophrenia, we performed linear regression analyses in which the reduction ratio of symptoms in each of the three domains (positive, negative, and disorganization) was the dependent variable. Loading scores of the GMV spatial components showing progressive changes (on the basis of paired *t* tests) were included as predictors, with levels of *F* to enter and *F* to remove set to correspond to *p* levels of 0.025 and 0.05, respectively. To correct for age, gender, and duration of illness and antipsychotic dosage (chlorpromazine equivalent), these variables were included as covariates. A Bonferroni corrected *p* < 0.05/3 = 0.016 was used for evaluating significance of the model.

### Symptom Severity at Baseline as a Predictor of Gray Matter Volume at Follow-Up

To assess whether baseline severity of symptoms predicted lower GMV after 1 year, we performed a series of linear regression analyses with loading scores of GMV components as the dependent variable. Symptom scores at the baseline were included as predictors, with levels of *F* to enter and *F* to remove set to correspond to *p* levels of 0.025 and 0.05, respectively. To correct for age, gender, and duration of illness and antipsychotic dosage (chlorpromazine equivalent), these variables were included as covariates. A Bonferroni-corrected *p* < 0.05/6 = 0.008 was used for evaluating significance of the model.

Given the chance of inflated type 1 error with stepwise models (30) for all significant results, we repeated the analyses by entering all predictors simultaneously and estimating the significance of regression coefficients at the threshold *p* < 0.05.

## Results

### Demographic and Clinical Characteristics

The demographic characteristics and clinical characteristics of the participants at baseline and at 1-year follow-up are shown in **Table 1**.

**Figure 1:**
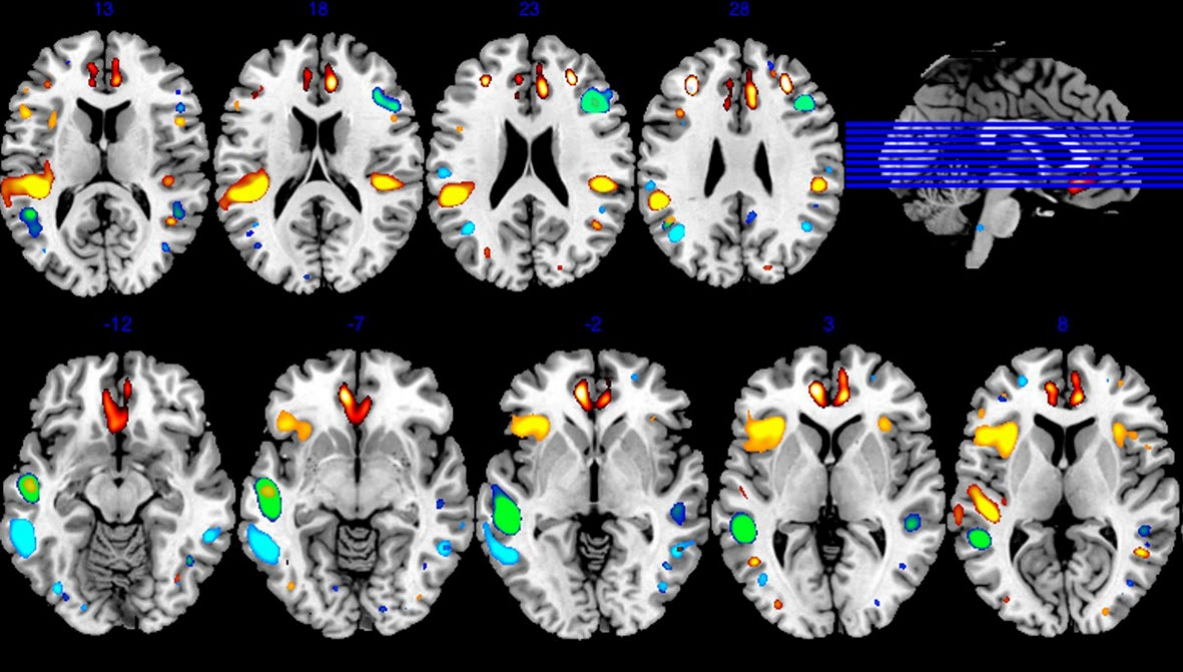
Six spatial components of gray matter decreased at the time point of 1 year. *p* < 0.005 corrected for Bonferroni multiple tests was deemed statistically significant.

The paired *t* tests revealed six components showing loading coefficients that significantly changed with time. The GMV for all of the six components decreased from the untreated first-episode baseline to 1-year follow-up time (**Supplementary Table 2**). This included Component 4 (C4), mainly consisting of ACC, medial frontal gyrus, cingulate gyrus, and middle frontal gyrus; Component 6 (C6), with parts of STG, insula, and inferior parietal lobule; Component 13 (C13), including inferior frontal gyrus, insula, and middle temporal gyrus (MTG); Component 15 (C15), including MTG and STG; Component 25 (C25), including precuneus, middle frontal gyrus, and MTG; and Component 30 (C30), including MTG, middle frontal gyrus, and inferior parietal lobule (**Table 2**, **Figure 1**, and **Supplementary Figure 1**).

**Table 2:** Anatomical description of the independent components.

Component number	Brodmann area	Volume (cc)	Max *Z* value (Talairach coordinates *x*, *y*, *z*) (left/right)
***Predominantly positive spatial components: 6 and 15***			
**Component 6**			
Superior temporal gyrus	13, 22, 39, 41, 42	6.1/1.3	11.2 (−41, −33, 16)/6.4 (46, −51, 11)
Insula	13, 40, 41	3.7/1.7	10.6 (−41, −33, 18)/7.5 (47, −28, 21)
Inferior parietal lobule	40	2.8/1.8	10.5 (−51, −38, 28)/8.1 (49, −29, 24)
Transverse temporal gyrus	41, 42	1.1/0.3	10.0 (−40, −30, 13)/5.1 (43, −26, 13)
Middle frontal gyrus	6, 9, 10, 46	1.0/0.4	7.4 (−30, −3, 44)/4.4 (44, 24, 22)
Middle temporal gyrus	20, 21, 37, 39	0.4/0.7	7.2 (−46, −56, 4)/5.9 (56, −47, −10)
**Component 15**			
Middle temporal gyrus	19, 21, 22, 37, 39	6.2/1.3	13.7 (−50, −28, −1)/5.6 (50, −35, 4)
Superior temporal gyrus	13, 21, 22, 39	4.5/2.5	13.5 (−50, −27, −1)/5.6 (49, −34, 4)
Middle frontal gyrus	6, 9, 10	1.5/0.6	8.9 (−37, 6, 40)/4.6 (35, −1, 42)
Supramarginal gyrus	40	1.0/0.0	6.9 (−45, −52, 32)/3.1 (53, −39, 33)
Precentral gyrus	3, 4, 6, 9, 13, 43	0.8/0.4	7.8 (−37, 6, 37)/4.7 (30, −20, 49)
***Predominantly negative spatial components: 4, 13, 25, and 30***			
**Component 4**			
Anterior cingulate	10, 24, 25, 32	5.4/5.8	6.4 (−9, 44, −1)/6.1 (9, 37, 16)
Medial frontal gyrus	6, 8, 9, 10, 11, 25, 32	4.4/5.4	6.1 (−7, 40, −7)/5.3 (9, 41, 14)
Cingulate gyrus	9, 24, 31, 32	3.6/3.3	5.0 (−7, 33, 29)/5.8 (9, 24, 26)
Middle frontal gyrus	6, 8, 9, 10, 46	2.7/2.4	8.2 (−31, 37, 24)/7.1 (30, 40, 22)
**Component 13**			
Inferior frontal gyrus	13, 44, 45, 46, 47	6.9/1.3	7.6 (−33, 24, 1)/5.9 (33, 24, 4)
Insula	13, 45, 47	3.5/1.2	7.7 (−32, 22, 3)/6.0 (33, 23, 4)
Middle temporal gyrus	21, 22, 39	1.0/0.1	5.7 (−52, −14, −8)/4.1 (56, −28, −14)
Precentral gyrus	6, 44	0.7/0.4	7.3 (−45, 19, 6)/5.4 (50, 11, 9)
Middle frontal gyrus	6, 8, 9, 10, 47	0.5/1.1	5.9 (−23, 23, 38)/7.2 (25, 30, 35)
**Component 25**			
Precuneus	7, 19, 31, 39	1.0/3.4	5.5 (−11, −63, 37)/11.1 (25, −58, 42)
Middle frontal gyrus	6, 8, 9, 10, 46	0.4/2.1	4.2 (−24, 50, 10)/18.4 (41, 24, 22)
Middle temporal gyrus	21, 37, 39	0.4/0.8	4.5 (−39, −61, 22)/5.7 (44, −59, 3)
Cingulate gyrus	31, 32	0.2/1.9	4.1 (−9, −40, 34)/6.0 (9, −45, 32)
Superior frontal gyrus	6, 8, 9, 10	0.1/1.0	4.4 (−23, 50, 10)/5.6 (23, 11, 45)
Inferior frontal gyrus	9, 13, 44, 45, 46	0.0/1.5	3.4 (−45, 21, 6)/9.5 (48, 22, 21)
Superior parietal lobule	7	0.0/1.2	−999.0 (0, 0, 0)/11.1 (25, −58, 43)
**Component 30**			
Middle temporal gyrus	20, 21, 22, 37, 39	6.6/3.4	12.5 (−55, −42, −8)/7.8 (51, −50, −2)
Middle frontal gyrus	6, 8, 9, 10	1.6/0.6	8.7 (−36, 13, 33)/5.5 (23, 36, 34)
Inferior parietal lobule	7, 40	1.0/0.7	5.8 (−51, −36, 39)/5.7 (42, −37, 57)
Inferior temporal gyrus	19, 20, 21, 37	0.7/0.4	7.4 (−52, −54, −3)/5.8 (42, −71, 2)
Middle occipital gyrus	18, 19, 37	0.7/0.4	6.0 (−37, −73, −7)/5.7 (42, −71, 3)
Postcentral gyrus	2, 3, 4, 5, 40, 43	0.6/1.4	5.8 (−55, −20, 22)/5.9 (56, −15, 30)
Precentral gyrus	3, 4, 6, 9	0.3/0.7	8.7 (−36, 13, 34)/5.8 (54, −17, 34)

The anatomical regions within each component are summarized after thresholding the z maps at z > 3.5 and volumes greater than 1 cm^3^.

### Baseline Predictors of Response to Treatment

Tests for multicollinearity indicated that a low level of multicollinearity was present (tolerance < 0.9 and variance inflation factor ≤ 2) for all predictors. The loading scores of C13 (higher baseline GMV of bilateral inferior frontal gyrus and anterior insula) predicted the improvement in positive symptoms as a single predictor (model *R* ^2^ = .125, *F* = 8.59, df = (1,60), *p* = 0.005). Higher baseline GMV of C13 also predicted improvement in disorganization symptoms (model *R* ^2^ = .179, *F* = 6.43, df = (2,59), *p* = 0.003) along with shorter duration of illness (*t* = −2.58, *p* = 0.012). None of the other five spatial components or covariates explained sufficient variance to be included in the final models predicting symptom reduction. When all predictors were simultaneously entered, C13 was the only component that predicted the reduction ratio of positive symptoms (β = −0.37, *t* = −2.9, *p* = 0.005) and disorganization symptoms (β = −0.28, *t* = −2.3, *p* = 0.03) (**Table 3**).

**Table 3:** Results of multiple regression with covariates entered simultaneously for positive and disorganization symptoms.

Reduction ratio	Positive symptoms		Disorganization	
Baseline GMV components	*β*	*t/p*	*β*	*t/p*
Component 4 (ACC)	0.261	1.57 (0.12)	0.084	0.52 (0.61)
Component 6 (STG)	−0.076	−0.56 (0.58)	−0.154	−1.16 (0.25)
Component 13 (Insula)	−**0.366**	−**2.90 (0.005)**	−**0.278**	−**2.26 (0.03)**
Component 15 (MTG/STG)	0.264	1.89 (0.06)	0.263	1.93 (0.06)
Component 25 (Precuneus)	0.022	0.17 (0.87)	0.12	0.95 (0.35)
Component 30 (MTG)	0.098	0.74 (0.47)	0.114	0.88 (0.39)

β: Standardized regression coefficient; ACC, anterior cingulate cortex; STG, superior temporal gyrus; MTG, middle temporal gyrus; GMV, gray matter volume.

The change in the severity of negative symptom after 1 year was not predictable on the basis of the loading coefficients of GMV components at baseline (**Supplementary Figure 3**).

### Symptom Severity at Baseline as a Predictor of Gray Matter Volume at Follow-Up

Tests for multicollinearity indicated that a low level of multicollinearity was present (tolerance ≤ 0.95 and variance inflation factor ≤ 1.5) for all predictors. None of the six GMV loading scores at 1-year follow-up were predicted by the baseline severity of positive, negative, and disorganization symptoms. The loading scores of C4 (indicating lower GMV of ACC and medial frontal gyrus) were predicted by older age (*t* = 3.96, *p* < 0.001) and female sex (*t* = 2.97, *p* = 0.004) but not by any of the three symptom scores at baseline (model *R* ^2^ = .26, *F* = 10.58, df = (2,59), *p* < 0.001). These results were unchanged even when the significance threshold for *F* to enter and *F* to remove was lowered to *p* = 0.05 and *p* = 0.1, respectively. We also did not find any significant results indicating a relationship between symptom severity at baseline and GMV at follow-up even when uncorrected *p* = 0.05 was used for evaluating the model significance.

## Discussion

Using a multivariate morphometric approach in a longitudinal MRI study of schizophrenia for the first time, we report three main findings: 1) the GMV of ACC, anterior insula, STG, MTG, dorsal lateral prefrontal cortex, precuneus, and inferior parietal lobule decreases over a 1-year period after commencing treatment for first-episode schizophrenia; 2) at the onset of first episode, the presence of lower GMV in bilateral inferior frontal gyrus and anterior insula predicts lack of a linear improvement in positive and disorganization symptoms despite antipsychotic treatment over 1 year; and 3) the presence of more severe symptoms during the first episode of psychosis does not indicate more pronounced gray matter reduction by 1 year. These observations highlight the prognostic utility of structural imaging, at least in the psychopathological domains of positive and disorganization symptoms following antipsychotic treatment.

One of the major limitations of the current study is the lack of a control group to establish the diagnostic specificity of GMV changes. But encouragingly, the spatial components showing significant GMV reduction over the first year of illness in our study are highly consistent with previous longitudinal morphometric studies in early stages of schizophrenia. In particular, spatial coordinate-based meta-analytic synthesis of stage-specific cross-sectional GMV changes in schizophrenia indicates a clustering of GMV reduction around anterior insula and ACC at early stages, with STG becoming involved at later stages (2). Progressive GMV reduction over 2–9 years of schizophrenia predominantly involves the lateral frontal (31–33), insula (26, 34–36), and ACC (26, 31), in addition to the STG (26, 31–33, 35–39), all of which showed GMV reduction in our study.

At the onset of psychosis, individuals that present with lower GMV in frontoinsula cortex (Component 13) respond poorly to treatment at 1 year. This finding is in line with Rosa et al. who reported pronounced GM reduction affecting left insula (and STG) in nonremitting first-episode schizophrenia (36), compared to those who achieve symptomatic remission. Structural alterations of anterior insula are well documented in schizophrenia (40–44). GMV reduction in anterior insula correlates with severity of positive symptoms (45, 46), as well as disorganization (43, 47) in individuals with schizophrenia. GMV of anterior insula is also associated with the quality of life measures in schizophrenia, highlighting the critical prognostic importance of this brain region. Our observations point to a possible mechanistic role for the anterior fronto-insula cortex and other regions included in Component 13 in the trajectory of positive and disorganization symptoms.

In contrast to the association between structural changes and positive/disorganization symptom domains, linear change in negative symptoms was not associated with any of the progressive gray matter alterations. This is not surprising, considering the elusive nature of the neuroanatomy of negative symptoms. We lacked measurements that could examine the two subdomains of negative symptoms—expressivity and motivation—limiting our ability to detect the specific structural relationships. In addition, the overall change in negative symptoms was much smaller in magnitude (see **Table 1**), when compared to positive and disorganization symptoms, reducing the likelihood of detectable structural changes occurring in association.

The severity of initial symptoms did not predict the GMV of any of the spatial components that show tissue reduction at year 1. Such a lack of association between illness severity and GMV reduction is not surprising, given previous similar negative observations (15, 48). Referring to McGlashan’s question (49) of whether active psychosis is neurotoxic, and subsequent observations by Andreasen et al. demonstrating an association between relapse duration (50) and antipsychotic dose (51), we note that putative neurotoxicity of a psychotic episode is unlikely to be due to higher severity of observed symptoms. As argued by Zipursky et al. (52), and recently shown by Moser et al. (53), factors such as alcohol and substance abuse and unhealthy lifestyle often associated with nonremitting symptoms confound the reports linking psychotic symptoms and GMV reduction (54, 55).

The strengths of the study included 1) the careful selection of the patients with antipsychotic-naive and first-episode schizophrenia, 2) the use of continuous measures of clinical improvement rather than a dichotomous approach that represents the actual structure of the symptom scores, and 3) the use of a data-driven and multivariate morphometry to analyze neuroimaging data to avoid hypothesis-based analyses that are limited to specific brain regions. There are several limitations, including the lack of control subjects, follow-up being limited to two observations, precluding examination of possible nonlinear effects of time, lack of antipsychotic switching data to assess treatment resistance, and the exclusion of a large number of treatment-seeking patients due to the inability to obtain informed consent or the presence of acute agitation. We also refrained from using machine-learning approaches to modeling, as we had a limited sample size.

We conclude that the gray matter structure of drug-naive patients at the onset of first-episode psychosis in schizophrenia carries valuable information regarding their 1-year clinical prognosis. This raises the possibility of investigating the utility of stratification based on morphometry, as a means to test the duration of long-term treatment that may be required after first episode of psychosis.

## Ethics Statement

This study was carried out in accordance with the Declaration of Helsinki and was approved by the Institutional Review Board of West China Hospital, Sichuan University. After a complete description of the study to the participants, written informed consent was obtained.

## Author Contributions

ML, LP, and TL designed this study. ML, XL, TD, WD, YL, LZ, XM, YW, HY, YM, and QW recruited the patients, administered assessment tools, and carried out data analysis. ML, XL, and LP wrote the manuscript. All authors listed have read, corrected, and approved it for publication.

## Funding

This work was partly supported by the National Nature Science Foundation of China Key Project (81630030 and 81130024 to TL); the National Natural Science Foundation of China/Research Grants Council of Hong Kong Joint Research Scheme (81461168029 to TL); the National Key Research and Development Program of the Ministry of Science and Technology of China (2016YFC0904300 to TL); the 1.3.5 Project for Disciplines of Excellence, West China Hospital, Sichuan University (ZY2016203 and ZY2016103); the Kilborn Fund for Internalization and the Bucke Family Fund, Schulich School of Medicine, University of Western Ontario (to LP); and the Opportunities Fund, Academic Medical Organization of South Western Ontario (to LP). ML was supported by a postdoctoral fellowship to undertake this work at the Robarts Research Institute under LP’s supervision.

## Conflict of Interest Statement

The authors declare that the research was conducted in the absence of any commercial or financial relationships that could be constructed as a potential conflict of interest.
