# Supplementary material for: Prognostic Utility of Multivariate Morphometry in Schizophrenia
Source: Front Psychiatry. 2019 Apr 15;10:245. doi: 10.3389/fpsyt.2019.00245 (PMC6476259; doi:10.3389/fpsyt.2019.00245)
Supplement: Supplementary file 1 [file DataSheet_1.docx]

**Supplementary Table 1. Number of patients and dosage for each type antipsychotic**

|  | Risperidone | | Olanzapine | Quetiapine | Aripiprazole | Sulpiride | Haloperidol | |
| --- | --- | --- | --- | --- | --- | --- | --- | --- |
| Number of patients | 32 | 8 | | 7 | 11 | 4 | 1 |  |

**Supplementary Table 2. Loading coefficients for the ICA Components showing significant changes at 1-year follow up among patients with schizophrenia**

| Component | LC baseline  Mean(SD) | LC 1-year  Mean(SD) | T / p value |
| --- | --- | --- | --- |
| C6 | 0.059(1.025) | -0.059(0.979) | 4.780/0.00029 |
| C15 | 0.070 (1.021) | -0.070 (0.981) | 5.905/0.0000 |
| C4 | -0.130(1.000) | 0.130(0.991) | -7.208/0.0000 |
| C25 | -0.078(1.005) | 0.078(0.997) | -6.822/0.0000 |
| C13 | -0.117(0.999) | 0.117(0.995) | -6.769/0.0000 |
| C30 | -0.124(1.038) | 0.124(0.953) | -5.971/0.0000 |

All results survived Bonferroni corrected p<0.005 statistical threshold. LC, loading coefficient; SD, Standard deviation; C, component number

The components of 6 and 15 were predominantly positive in loading, and their loading coefficients reduced at follow up compared to the baseline, indicating reduction in GMV with time. The components of 4, 13, 25, 30 were predominantly negative, with the loading coefficients that increased at follow up compared to the baseline, indicating reduction in GMV with time.


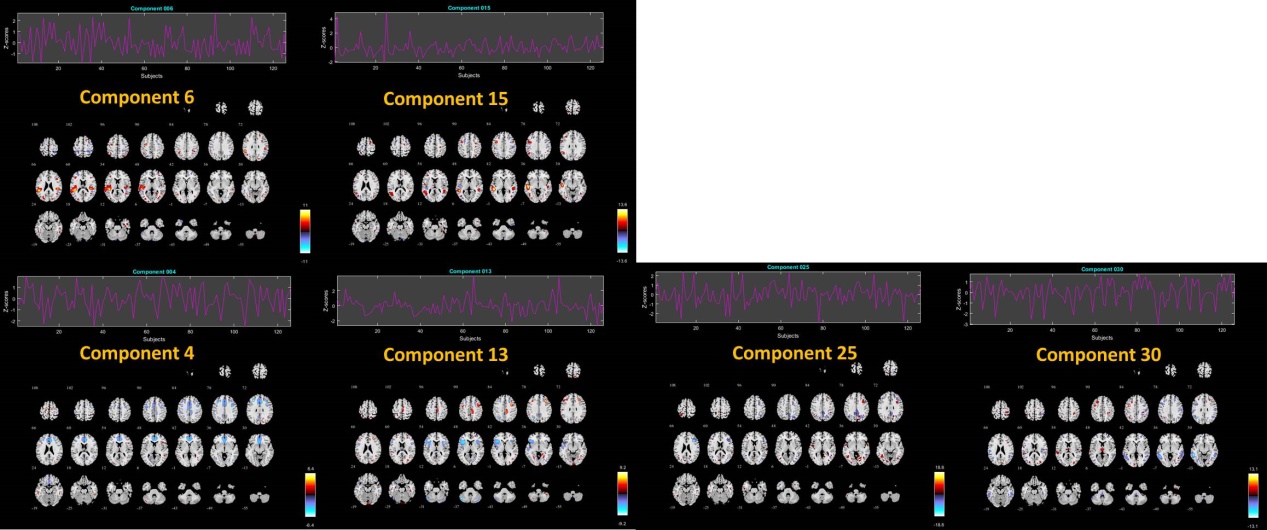


**Supplementary Figure 1.** 6 spatial components of gray matter decreased from baseline to 1-year follow-up (Bonferroni corrected P<0.005 for paired t tests). The components of 6 and 15 were predominantly positive, while the components of 4, 13, 25, 30 were predominantly negative in loading coefficients. Gray matter volume decreased with time across all 6 components.


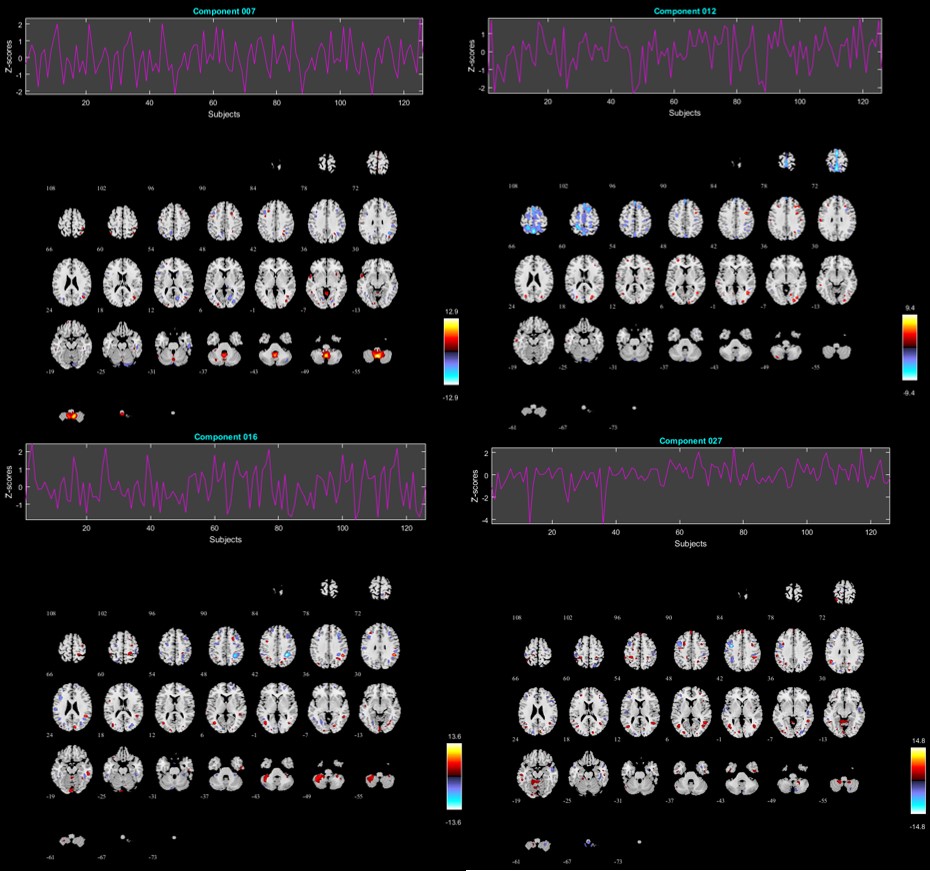


**Supplementary Figure 2.** 4 components identified as obvious artifacts

**Supplementary Figure 3.** Scatter plots of Component 13 loading scores and adjusted reduction ratio scores indicating linear symptom change over 1 year. Higher values of C13 loading scores indicate lower grey matter volume in insula, inferior frontal gyrus and other regions shown in Table 2. Higher adjusted values of reduction ratio indicate greater symptomatic symptom from baseline to 1-year time point.
